# Pathobiology of Hemangiosarcoma in Dogs: Research Advances and Future Perspectives

**DOI:** 10.3390/vetsci2040388

**Published:** 2015-11-06

**Authors:** Jong-Hyuk Kim, Ashley J. Graef, Erin B. Dickerson, Jaime F. Modiano

**Affiliations:** 1 Department of Veterinary Clinical Sciences, College of Veterinary Medicine, University of Minnesota, St. Paul, MN 55108, USA; E-Mails: ajgraef@umn.edu (A.J.G.); edickers@umn.edu (E.B.D.); modiano@umn.edu (J.F.M.); 2 Animal Cancer Care and Research Program, University of Minnesota, St. Paul, MN 55108, USA; 3 Masonic Cancer Center, University of Minnesota, Minneapolis, MN 55455, USA; 4 Stem Cell Institute, University of Minnesota, Minneapolis, MN 55455, USA; 5 Center for Immunology, University of Minnesota, Minneapolis, MN 55455, USA

**Keywords:** animal model, dog, hemangiosarcoma, tumor microenvironment

## Abstract

Hemangiosarcoma (HSA) is an aggressive and common cancer in dogs. While cutaneous masses are often treatable by tumor excision, visceral tumors are almost always incurable. Treatment advances for this disease have been limited due to a poor understanding of the overall tumor biology. Based upon its histological appearance, HSA has been presumed to originate from transformed endothelial cells; however, accumulating data now suggest a pluripotent bone marrow progenitor as the cell of origin for this disease. More recently, the identification of a novel subclassification of HSAs has provided a foundation to further our understanding of the cellular characteristics of HSA tumor cells, along with those of the cells comprising the tumor microenvironment. These discoveries hold promise for the development of new approaches to improve treatments for canine HSA, as well as to establish the utility of this disease as a spontaneous model to understand the pathogenesis and develop new treatments for vascular tumors of humans. In this review, we will provide a brief historical perspective and pathobiology of canine HSA, along with a focus on the recent advances in the molecular and cellular understanding of these tumors. In addition, future directions that should continue to improve our understanding of HSA pathogenesis will be discussed.

## 1. Introduction

Diligent clinical research over the past 50 years has allowed veterinarians to adapt or develop protocols to treat companion animals with cancer, providing pet owners reasonable options ranging from palliative care to therapies with curative intent. In addition to adapting protocols using conventional modalities (surgery, radiation, and cytotoxic chemotherapy), targeted drugs and immunotherapy approaches have been developed specifically for the veterinary market [1,2]. However, it could be argued that basic research has lagged behind these clinical advances. In particular, issues such as tumor heterogeneity in some of the most common cancers we diagnose have yet to be addressed, and advances to treat highly aggressive tumors such as hemangiosarcoma (HSA) have been modest. It is only recently that these have become areas of emphasis in veterinary cancer research, shifting the focus toward improving our understanding of cancer pathogenesis, developing stratification schemes that can improve prognosis and prediction, and applying the knowledge gained to advance innovations in therapy. Here, we will present a brief history and an overview of the pathobiology of canine HSA, along with a focus on scientific advances in recent research; specifically, we will emphasize several discoveries that have improved our understanding of the molecular pathogenesis of this disease and that are providing a new foundation for future studies and therapeutic intervention.

## 2. Historical Perspective of Canine HSA

In humans, angiosarcomas are very rare; they account for 0.01%–0.1% of all human cancers, although they are extremely aggressive. Angiosarcomas were initially reported as a pathological entity in people exposed to thorium or vinyl chloride after latency periods of 20 to 30 years [3,4]. The first reports of canine HSA in the biomedical literature are contemporary to these findings, dating to the 1950s and 1960s [5,6,7,8] and coinciding with the time when the relationship between dogs and humans had started to evolve from one where dogs largely served working roles such as herding, guarding, and hunting to one where they were considered members of the family. By the mid-1970s, it was apparent that the incidence of HSA in dogs was 25 to 100 times greater than the incidence of angiosarcoma in humans, and breed predilections for this disease were clearly documented in Europe and in the United States by the end of the 1980s [9,10,11,12]. In 1980, Priester and McKay reported breed-associated risks for development of hematopoietic neoplasms, including HSA, based on data from the Veterinary Medical Databases (VMDB). Breeds that represented the highest risk included Boxers, Basset hounds, and St. Bernards. Scottish terriers, Bulldogs, Airedales, Weimaraners, golden retrievers, Doberman pinschers, Labrador retrievers, English setters, and Great Danes were among other breeds at risk, whereas mixed breeds, miniature and toy poodles, Pomeranians, Chihuahuas, Boston terriers, cocker spaniels, and dachshunds were considered less likely to develop these tumors (relative risk < 1) [13,14]. More recent information regarding hematopoietic cancers in dogs obtained from health surveys privately commissioned by American Kennel Club National Breed Clubs suggests the prevalence of HSA in the US might be changing [15]. That is, the prevalence in breeds considered to be at high-risk could be different from past reports, possibly due to firm embedding of heritable risk traits by changes in breed popularity or by popular sire effects.

The causative factors of angiosarcoma in humans also seem to have evolved over the last 40–50 years, with most contemporary patients having no history of occupational or therapeutic exposure to known risk factors [16,17], likely because of regulation created from a deeper understanding of the link between DNA-damaging agents and angiosarcoma.

## 3. Classical Pathology of Canine HSA

**Figure 1:**
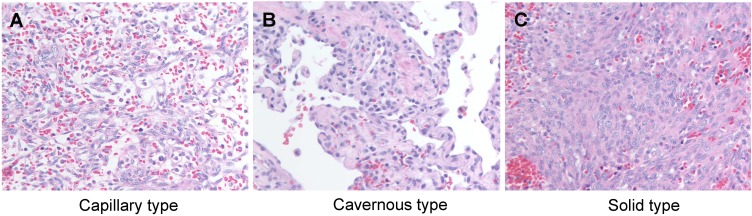
Histological appearance of canine hemangiosarcoma. (**A**) Capillary type; (**B**) Cavernous type; (**C**) Solid type. H&E stain. Magnification = 400×.

HSA is historically classified as a vascular tumor, and specifically, as a tumor arising from malignant endothelial cells. Histologically, the tumors are cellular with moderate to extensive areas of hemorrhage and necrosis. Morphologically, they can have capillary, cavernous or solid appearance (Figure 1), and the malignant cells can be highly pleomorphic with features that are reminiscent of those seen in other sarcomas. The main distinction is that tumor cells are observed lining irregular vascular spaces (capillaries or sinusoids) filled with blood, and they express proteins commonly associated with endothelial differentiation [18]. The histologic classification of these tumors has long been a source of controversy. While debate has raged on about other tumors regarding subclassification according to morphology or topology, “HSA” is a diagnostic monolith that describes tumors with diverse histologic properties associated with vascular structures in numerous organs.

HSA can develop in any tissue or organ containing vascular structures. The most common primary sites for HSAs include spleen, right atrium of the heart, subcutis, and dermis. Visceral HSAs are more common than cutaneous HSAs, and they are associated with poorer prognosis [19]. However, the morphology, histological organization, and pathobiological features are indistinguishable among visceral forms of the disease that originate from different anatomical locations [20]. The pattern of growth involves infiltration into normal tissues surrounding the tumor as well as distant metastasis. Yet, the disease is potentially insidious; that is, the rate of growth in the early stages appears to be relatively slow, and this finding that is recapitulated in xenograft models [21,22]. Dogs harboring even large HSAs may show no clinical signs or evidence that they have a life threatening disease.

Generally, tumor-associated blood vessels are tortuous and malformed, and blood cells tend to pool in them and clot [23]. The clots prevent blood and nutrients from reaching tumor cells, in turn causing these cells to die. This creates small ruptures in the tumor through which blood may escape into the abdomen, pericardial sac, pleural cavity, or subcutaneous space [11]. Depending on the amount of blood loss, affected dogs may show constitutional signs, including lethargy and weakness, but these are transient and resolve as dogs reabsorb the blood components and make new blood cells.

The histological appearance and the progression of the disease in humans with angiosarcoma resemble those seen in dogs [24]. More than half of the patients with this disease die within the first year of diagnosis and tumor related mortality is extremely high [25].

## 4. Ontogeny of Canine HSA

The etiologic and cellular origins of HSA are incompletely understood. Breed predilection remains a consistent finding, suggesting that heritable traits contribute to this disease [26,27]. Even so, the median age at diagnosis, which is approximately 10 years and represents animals of advanced age, has not changed over the past five decades. The contribution of heritable traits, while important, is not singularly responsible for this disease; a recent study suggests that the number of tissue-specific stem cell divisions can explain the risk for most human cancers [28]. It has been proposed that this is directly proportional to the error rates of DNA polymerases, adding an important stochastic component to the risk that can be attributed to heritable factors [29].

Environmental factors also are likely to play a role in the etiology of HSA, although risk factors to which a realistic contribution could be attributed based on exposure and disease have not been identified in pet dogs. Exposure to high levels of ionizing radiation can promote development of HSA in experimental dogs [30,31,32,33]. Infectious etiologies have not been conclusively identified in this disease, although an association between HSA and Leishmaniasis was reported in three dogs in one study [34], and another reported a statistically increased frequency of *Bartonella spp*. DNA in samples from splenic canine HSA as compared to spleens from specific pathogen-free dogs [35]. Overall, the relatively high frequency of HSA in dogs might be due to anatomic or functional features of the canine microvasculature or of canine inflammation and hemostasis.

**Figure 2:**
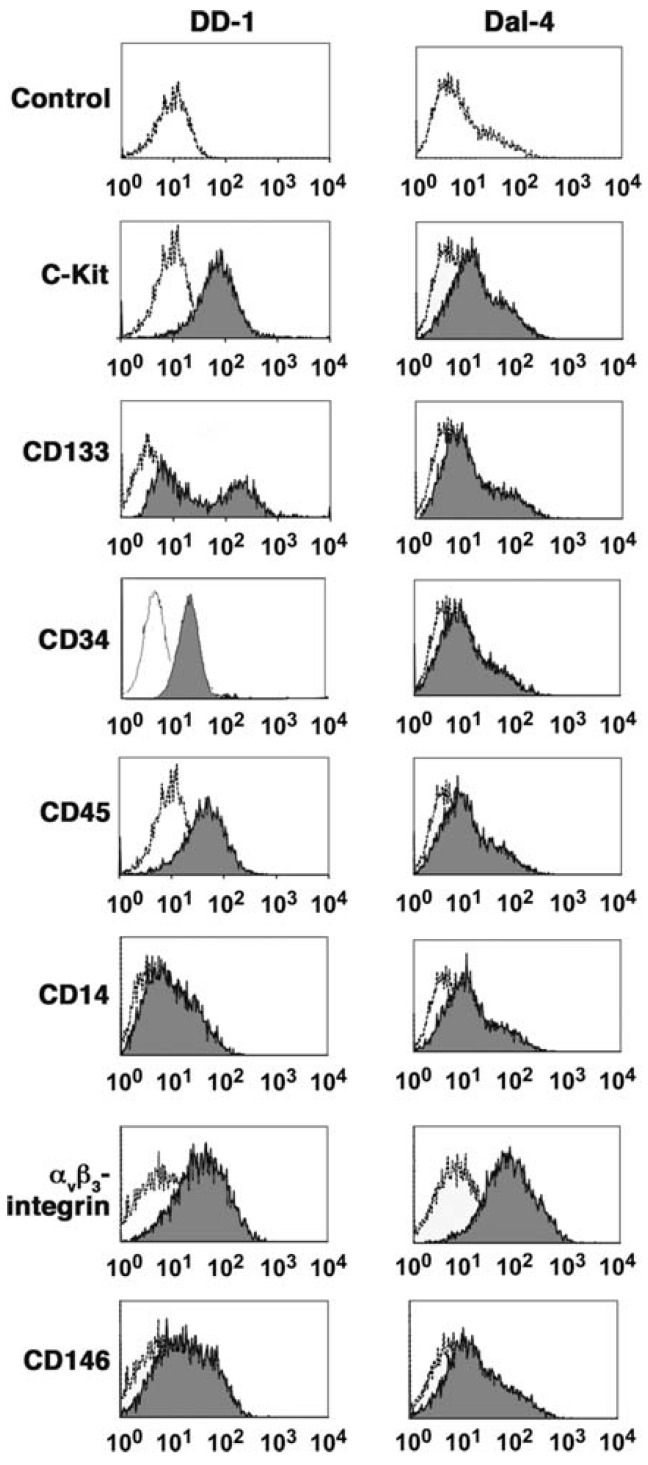
Expression of cell-surface determinants in canine HSA cell lines. One-dimensional overlay histograms show canine HSA cells stained with the indicated antibodies and analyzed by flow cytometry (gray). X-axes represent logarithmic fluorescence intensity and y-axes represent cell numbers (5000 to 10,000 events analyzed). Histograms are overlaid on corresponding negative controls using irrelevant antibodies (white). Data shown for DD-1 cells are from three experiments and represent more than seven experiments done. Data for Dal-4 cells represent more than four experiments done. Variability in antigen expression by these cells is described in the text. Reproduced with permission from [39].

While the etiology of the disease remains uncertain, the cellular origin of HSA is becoming clearer. It was originally presumed that HSAs originated from transformed endothelial cells; this presumption was based largely on the histological appearance of these tumors. However, data from the past 10 years implicate a pluripotent bone marrow progenitor as the cell of origin for this disease [17,20,36,37,38,39,40]. These data come from analysis of HSA-derived cell lines, which are representative of disease anatomy and breed diversity. Studies using cell lines have provided a clearer picture of HSA cell biology including characteristics of cell surface antigen expression [18,39], tumor suppressor gene inactivation [41], and the generation of tumor cell genome-wide gene expression profiles without interference from tumor-associated stroma [20,36,40]. An important aspect arising from these data was the observation that HSA cells in isolation (cell culture) have phenotypic properties suggestive of bone marrow ontogeny and specifically of bone marrow derived stem cells [39] (Figure 2). Alone, these data could not distinguish if the tumors themselves arose from a cell in the bone marrow that migrated to a vascular plexus, or if they originated from a bona fide stem cell. Gene expression profiling data offered some clues, however, since the recurrent signature of these genes was associated with enrichment of angiogenic and pro-inflammatory genes, with no evidence of tissue specificity [40].

**Figure 3:**
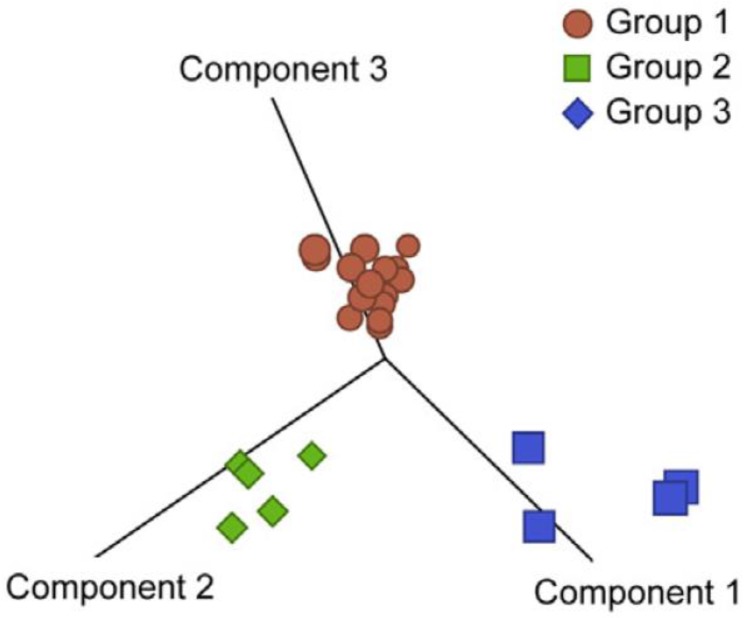
Genome-wide expression analysis identified three molecular subtypes in hemangiosarcoma. Genes with variance >0.5 across 24 samples were used to generate principal component analyses. Samples were assigned to one of three groups by unsupervised clustering to identify genes with significantly different expression between groups (analysis of variance *p* < 0.001 and an average fold change >3 between groups). Reproduced with permission from [20].

More recent data corroborated these findings and also showed that HSAs could be stratified according to the expression of pro-inflammatory genes (inflammatory subtype), endothelial cell-matrix interaction genes (vascular or angiogenic subtype), and pro-adipogenic and connective tissue-forming genes (adipogenic subtype) [20] (Figure 3). These genes also reflected the composition of the tumor microenvironment, and potentially the balance of hypoxia and inflammation during tumor formation [17,42].

While the same depth of ontogenetic molecular studies has not been achieved in human angiosarcoma, the data suggest that this disease can also arise from similar multipotent, bone marrow-derived progenitor cells or early endothelial progenitor cells [43,44], while late endothelial progenitor cells or differentiated endothelial cells are thought to be a cell of origin for human hemangioma [43]. But the events that drive progression of angiosarcoma are incompletely understood. There is debate regarding the role of common pathways of tumorigenesis in this disease [25]; for example, previous studies identified *RAS* mutations, *KDR* (kinase insert domain receptor, also known as VEGFR2) mutations, *MYC* gene amplification, and alterations in the p53, CDKN2, NF-κB/IL-6, MAPK, and PIK3CA/AKT/mTOR pathways in angiosarcoma [21,24,45,46,47,48]. However, the studies represent small case series, *RAS* mutations appear to be almost exclusively seen in tumors caused by exposure to vinyl chloride, and the significance of other abnormalities is unclear.

In dogs, cell intrinsic abnormalities include mutations of *PTEN* [41], altered expression of angiogenic factors [49], activation of tyrosine kinase pathways [50,51,52] or Akt/mTOR/4E-BP1 pathways [53,54], and copy number aberrations involving *CDKN2A*, *VEGFA*, and the *SKI* oncogene [55]. The importance of the PIK3CA/AKT pathways in angiosarcoma is evident based on the conserved association between abnormalities in this pathway and the appearance of HSA or angiosarcoma across dogs, humans, and zebrafish, three evolutionarily distant species. In humans, activation of PIK3 seems to occur through different mechanisms depending of the topography of the tumors, with deficiencies of *PTEN* seen more frequently in bone angiosarcomas and overexpression of *KIT* seen more frequently in soft tissue angiosarcomas [56]. However, a spontaneous mutation of the *PTEN* C2 domain resembling those seen in dogs also has been reported in a liver angiosarcoma from a human patient [57]. Finally, angiosarcomas also are a consequence of *PTEN* haploinsufficiency in zebrafish [58].

**Figure 4:**
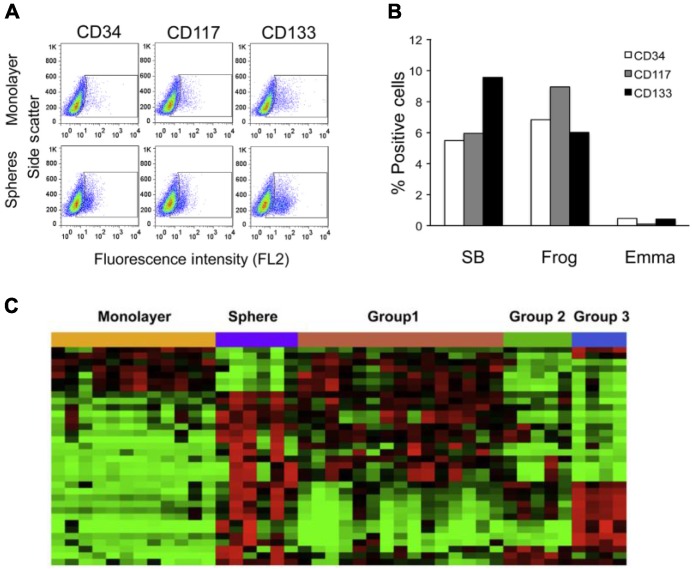
Sphere cells serve as an *in vitro* model for HSA progenitor cells and express markers for endothelial and hematopoietic progenitors. (**A**) Flow cytometric analysis of expression of CD34, CD117, and CD133; (**B**) Graphical representation of the percentage of positive cells for each marker in the monolayer cells subtracted from the percentage of positive cells detected in the corresponding sphere cells. Results show a relative enrichment of the markers in sphere cells; (**C**) Heat map of 34 gene expression patterns significant in both the comparisons between the genes differently expressed in intact tumors and the genes differently expressed by spheres. For (**C**), each data set was independently mean centered. Reproduced with permission from [20].

The inability to identify universal, cell intrinsic abnormalities in HSA is probably because these tumors are heavily reliant on their microenvironment for survival. HSA cells respond to cues from the microenvironment, and they adopt various functions as part of this response [20]. These functions include not only the potential to form anatomically distinct structures or tumors, but also the potential to direct other cells in their environment to do so. HSA cell lines include subpopulations (established from an *in vitro* sphere cell model) (Figure 4A,B) that retain traits associated with “cancer stem cells”, including self-renewal, chemoresistance, and increased tumorigenicity *in vivo* [20,59,60]. Each tumor may achieve these properties by independently disabling or enabling molecular networks associated with self-renewal and survival (Figure 4C). Although much work remains to be completed, it is apparent that some or many properties of HSA are dependent on interactions between tumor cells and their local microenvironment. These interactions probably depend on the microanatomy of the niche and the capability of the cells to alter the niche by recruitment or reprogramming of local stromal cells [22]. Local or systemic microangiopathy may precede the disease, but it also may be a consequence of the vascular disruption it creates. Platelets and platelet derived factors, inflammation, and hypoxia are probably key drivers of the disease.

## 5. HSA and the Tumor Microenvironment

**Figure 5:**
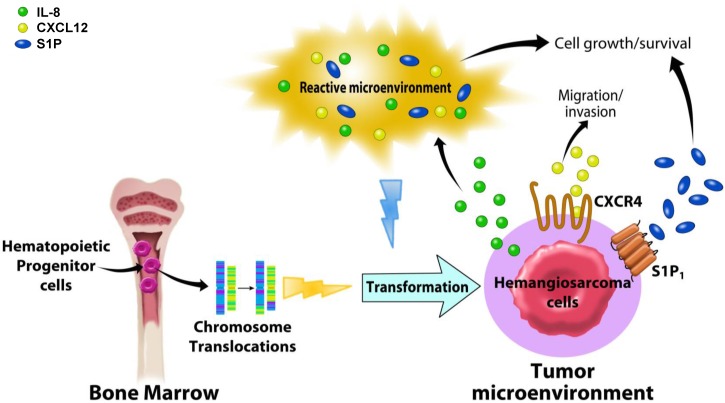
Overview of the relationships between biological signaling molecules and the tumor microenvironment in canine hemangiosarcoma. Canine hemangiosarcoma is thought to originate from hematopoietic progenitor cells in the bone marrow. Chromosome translocations and a reactive microenvironment are suggested as potential genetic and biological events that may transform the hemangiosarcoma progenitor cells. Interleukin (IL)-8, produced by hemangiosarcoma cells, is thought to modulate the tumor microenvironment, promoting the growth and survival of tumor cells. CXCR4 and its ligand, CXCL12, found to be abundant in hemangiosarcoma tissue, transduce biological signaling, causing tumor cells to increase their motility to migrate and invade into the other sites for metastasis. Canine hemangiosarcoma cells that consume Sphingosine-1-phosphate (S1P) from the tumor microenvironment induce intracellular signaling through S1P receptor-1 (S1P_1_), increasing cell growth and survival. It is suggested that these chemokines and modified biolipids are key regulators for hemangiosarcoma behavior, and their signaling pathways are potential therapeutic targets.

Bidirectional interactions between malignant cells and their local environment are key regulators of tumor growth and progression. Recent studies have focused on understanding how specific components of the HSA tumor microenvironment enhance tumor growth and survival or promote migration of tumor cells. Inflammation and angiogenesis are recurrent features of HSA cells [20,40] and these cells have been shown to express functional receptors that initiate biologically relevant signals upon binding to chemokines including interleukin (IL)-8 and chemokine (CXC motif) ligand-12 (CXCL12, also known as stromal cell-derived factor-1α or SDF1α), and modified sphingosines [22,40,61,62] (Figure 5).

IL-8 is a well-known pro-inflammatory cytokine that plays a pivotal role in inflammation and angiogenesis; thereby, it has been robustly studied as a key regulator of tumor microenvironment [63,64]. IL-8 transduced biological signals in HSA cells, inducing calcium mobilization [22] and altering transcription of its own gene by what appeared to be a negative feedback loop [65]. A similar negative feedback loop seemed to control the mRNA levels of Slug (Snail-2), a transcription factor associated with stem cell maintenance. Curiously, IL-8 did not directly promote HSA cell proliferation or survival in culture, but its gene expression was associated with gene signatures reflecting reactive tumor microenvironments [22]. The signature was observed in tumor tissues and in cell lines, indicating tumor autonomous effects were dominant and suggesting that IL-8 production is part of an adaptive mechanism used by HSA cells to modulate their microenvironment. This finding was illustrated by a mouse xenograft model where IL-8 blockade inhibited HSA cell survival and engraftment [22].

CXCL12 and its receptor chemokine (CXC motif) receptor-4 (CXCR4) play a role in migration of hematopoietic stem cells and cancer cells [66,67,68]. Their interaction leads cancer cells to move toward metastatic sites that consist of CXCL12-enriched microenvironments [69]. In canine HSA, gene expression profiling identified gene signatures of hematopoietic functions and cell migration based upon the expression of CXCR4 and CXCL12 [61]. Moreover, the hematopoietic gene signature strongly supported the premise that HSAs arise from hematopoietic precursors, as discussed above. The potential importance of the CXCR4/CXCL12 signaling axis in metastasis of HSA cells was further emphasized by the fact that cells expressing CXCR4 showed increased migration and invasion upon exposure to CXCL12, and this was inhibited by plerixafor (AMD3100), a CXCR4 antagonist.

A role for CXCR4 signaling in HSA metastasis is possible, but the origin of CXCL12 as an autocrine, paracrine or endocrine factor, as well as the role of CXCR4 expressed in inflammatory and stromal cells that form the tumor niche remain to be determined. Furthermore, it is unclear whether HSA cells create CXCL12-enriched environments that affect CXCR4-expressing cancer cells, or whether these CXCR4-expressing cells migrate to and/or colonize organs and tissues where there is abundant CXLC12. These questions will need to be answered in order to determine if modulation of the CXCR4/CXCL12 axis has therapeutic potential for canine HSA and for human angiosarcoma.

The sphingosine-1-phosphate (S1P) receptor-1 (S1P_1_), which is the product of the endothelial differentiation gene-1 (EDG1), is a member of the S1P receptor family [70]. S1P is a bioactive lipid that counteracts the pro-apoptotic effect of ceramides; S1P receptor signaling is important to maintain immune and vascular homeostasis, but it also has been shown to contribute to pathologic cell growth and cancer [71,72] making components of this axis potential therapeutic targets [72,73]. As is true for CXCL12, the source of S1P and the importance of its effects on tumor stroma remain to be established [72]. A recent study demonstrated that production of S1P by cultured HSA cells was negligible [62], suggesting that elements in the tumor microenvironment such as erythrocytes and platelets are more likely to be the sources of this molecule [62]. The importance of S1P signaling in HSA was further established by the observation that FTY720, a structural analog of S1P that promotes degradation of S1P_1_ [72], induced apoptosis of HSA cells [62]. The Food and Drug Administration has approved FTY720 as an immunosuppressant, suggesting that compounds that attack this pathway could have favorable toxicity profiles and could be useful in the treatment of canine HSA and human angiosarcoma.

**Figure 6:**
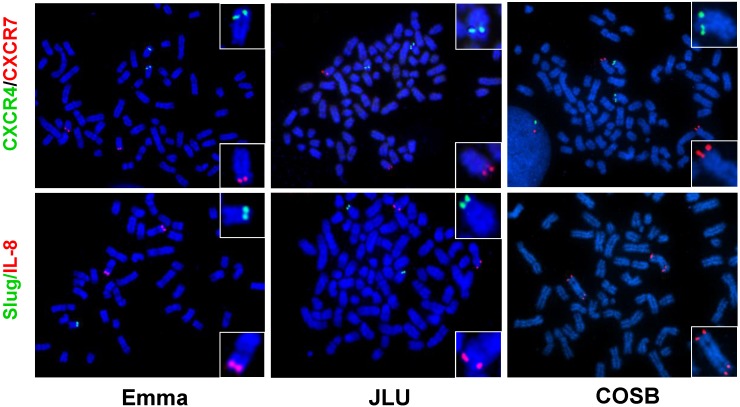
Cytogenetic appearance of representative canine hemangiosarcoma cells. *CXCR4*, *CXCR7*, *SLUG*, and *IL-8* genes were analyzed by fluorescence *in situ* hybridization (FISH) using BAC clones (CHORI-82). (Upper panel) Green and red spots indicate CXCR4 (CHORI-82-112B08) on CFA 19 and CXCR7 (CHORI-82-52B03) on CFA 25, respectively. (Bottom panel) Green and red spots indicate Slug (CHORI-82-130F20) on dog chromosome (CFA) 19 and IL-8 (CHORI-82-187A21) on CFA 13, respectively. Emma, JLU, and COSB = HSA cell lines.

Interactions between canine HSA cells and non-cellular constituents of the microenvironment, especially hyaluronic acids, also appear to have a role in maintaining the inflammatory and angiogenic niche. It is not clear if molecules that modulate the microenvironment in HSA are frequently deregulated in the tumor cells themselves. To address this, we used fluorescence *in situ* hybridization (FISH) to evaluate copy number abnormalities for the genes encoding *IL8*, *CXCL12*, *CXCR4*, *CXCR7* (an alternative CXCL12 receptor), *CD44* (a hyaluronic acid receptor), and *SLUG* in four HSA cell lines. Our results show that *CXCR4* and *CXCR7* appear to be cytogenetically stable, and that *IL8* is duplicated as part of centromeric fusions of canine chromosome (CFA) 13 and there seems to be copy number loss of *SLUG* in some tumors (Figure 6). The degree of aneuploidy for *CD44* and *CXCL12* was variable, showing no consistent patterns of copy number gains or losses among the four cell lines examined.

Yet, copy number abnormalities were not directly proportional to IL-8 or CXCL12 production, as measured by steady state levels of gene expression or protein secretion [22,61], suggesting that these molecules are part of complex interacting networks and not unique drivers for this disease. Ongoing work to define the role of these pathways using *in vivo* systems will provide a better understanding on how modulating the tumor microenvironment affects the progression of HSA, as well as how these pathways might influence progression of human angiosarcoma.

## 6. Future Directions

### 6.1. Establishment of Xenograft Models

**Figure 7:**
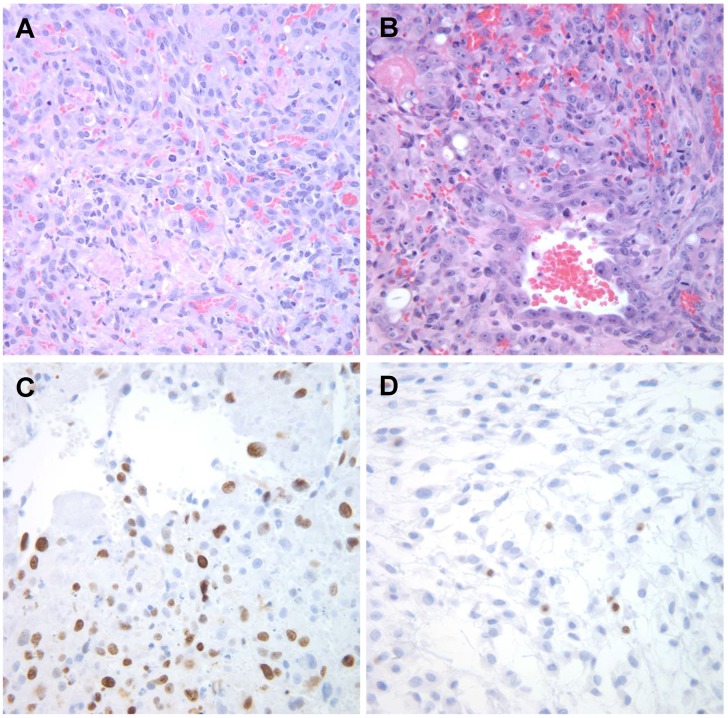
Microscopic appearance of SB-HSA canine hemangiosarcoma xenografts. (**A** and **B**) Photomicrographs showing examples of tumors from two mice inoculated with 5 × 10^6^ SB-HSA cells stained with H & E; (**C**) Immunostaining of the tumor in *a* for Ki-67 using antibody MIB-1 (recognizes canine Ki-67); and (**D**) antibody TEC-3 (recognizes murine Ki-67). Magnification = 400×. Reproduced with permission from [22].

Tumor xenografts represent a convenient means to evaluate preclinical outcomes and to test novel, potential anticancer drugs *in vivo* [74]. The transplantation of primary tumor samples from pet dogs into mice has been increasingly reported: *i.e.*, for canine mammary carcinomas [75,76], lymphoma [77], melanoma [78], osteosarcoma [78,79]. Likewise, canine HSAs are adaptable and grow in immunocompromised mice [21,22,54,80,81] (Figure 7); however, protocols are not yet standardized among xenograft models, and data are inconsistent regarding tumor penetrance, serial transplantation potential, latency of tumor development, and growth rates in models reported by different laboratories. These create a barrier in the development of effective therapies for canine HSA, since reliable results have not yet been obtained using this approach. Therefore, advances in xenograft implantation and elimination of inconsistencies among model systems are pressing needs in HSA research. To date, our lab has used various approaches to generate xenograft models of canine HSA, including patient-derived tumor xenografts (PDTX) and implantation of HSA cell lines cultured *in vitro* with and without supporting extracellular matrices that aid in remodeling the tumor microenvironment.

### 6.2. Chromosomal Translocations

The diverse cellular ontogeny and heterogeneity of sarcomas has provided an impetus to develop molecular approaches to classify these tumors [82,83]. This has uncovered two categories of human sarcomas: one consisting of tumors with simple genetic alterations, and the other comprising sarcomas with complex, chaotic karyotypes [82,84]. Most human sarcomas with simple genetic aberrations are translocation-related cancers [85]. Human angiosarcomas fall into the category of sarcomas with complex karyotypes [46,84]. A genetic categorization based on cytogenetics has not been described for canine HSA, and driver events for this tumor still need to be rigorously defined and validated.

In addition to providing an unbiased and comprehensive assessment of genome-wide gene expression in canine HSA [20,40], the discovery of novel transcripts resulting from fusion genes (*i.e.*, chromosome translocations) is a relevant approach to unveil the precise tumorigenic mechanism as well as to define pathogenesis [86,87]. We recently generated a dataset of next generation, deep RNA-Seq from 62 cases of spontaneous canine HSA, to identify chromosome translocations. The gene fusions that we discovered seem to be associated with the different HSA molecular subtypes, allowing us to consider development of specific targeted therapies to attack this disease. In this regard, identifying chromosome translocations will be clinically relevant and contribute to unveiling the pathogenesis of HSA. Although the ongoing discovery of fusion genes and their pathogenic significance is in the early stages of development, advancing our understanding of the molecular mechanisms of canine HSA, and potentially of human angiosarcoma, remains a likely outcome.

## 7. Conclusions

In this review, we have presented the historical views and the pathobiology of canine HSA and its relationship to human angiosarcoma. New discoveries regarding the ontogeny and the biology of HSA and its microenvironment will help shift the paradigm to develop effective strategies to control, treat, and ultimately prevent this disease.
